# Transient Intraperitoneal Residence of *Dirofilaria immitis* Larvae in the Mongolian Gerbil (*Meriones unguiculatus*)

**DOI:** 10.3390/pathogens15020199

**Published:** 2026-02-10

**Authors:** Elyssa Campbell, Michael Dzimianski, Katelin Greenway, Kaori Sakamoto, Andrew Moorhead

**Affiliations:** Department of Infectious Diseases, College of Veterinary Medicine, University of Georgia, Athens, GA 30602, USA; ejacob7@uga.edu (E.C.); ksakamo2@ncsu.edu (K.S.)

**Keywords:** *Brugia malayi*, cellular immune response, *Dirofilaria immitis*, host specificity, host–parasite interaction, larval development, larval sequestration, Mongolian gerbil, peritoneal cavity

## Abstract

Understanding the determinants of host specificity in *Dirofilaria immitis* can be advanced through the use of the nonpermissive Mongolian gerbil (jird) model. We hypothesized that host immunity dictates *D. immitis* establishment following the third larval molt. Jirds were infected intraperitoneally with 100 *Brugia malayi* (permissive control) or *D. immitis* third-stage larvae (L3). Necropsies occurred at 1, 3, 10, and 36 days post infection (dpi) to quantify larvae via peritoneal lavage. Initial recovery at 1 dpi showed 37.4% for *B. malayi* but only 0.4% for *D. immitis* (*p* < 0.0001). *Dirofilaria immitis* recovery increased to 23.6% by 3 dpi, suggesting a period of transient tissue residence during the third molt. Recovery for both species decreased by 10 dpi. *Brugia malayi* reached the immature adult stage (15.2%) by 36 dpi, whereas no viable *D. immitis* were recovered (*p* < 0.0001). These findings suggest that *D. immitis* larvae encounter a robust cellular response, primarily macrophages, shortly after the third molt. Identifying the specific larval stage at which establishment fails provides critical insight into the mechanisms governing filarial host specificity.

## 1. Introduction

Canine heartworm disease, caused by the filarial nematode *Dirofilaria immitis*, was diagnosed in around 188,000 dogs in the United States in 2025 [1]. These parasites are transmitted by mosquito vectors that ingest blood containing microfilariae (MF) from a heartworm-infected dog. The MF undergo two molts in the mosquito, developing to third-stage larvae (L3). These L3 are infectious, as they have made their way to the head, specifically the proboscis of the mosquito. During the mosquito’s next blood meal, the L3 are deposited on the dog’s skin and enter the bite wound to migrate through the cutaneous tissue and molt into fourth-stage larvae (L4). The L4 travels through the tissues and molts one last time into immature adults (IA) between 50 and 58 days post infection [2]. As early as 68 days post infection [3], the IA establish in the heart and pulmonary arteries, becoming sexually mature adults at around 4.5 months post infection and producing MF six months post infection [3]. Adult *D. immitis* worms can live between 5 and 7 years in dogs [2] and can cause irreparable clinical and pathological changes to the heart and pulmonary artery, including inflammation, pulmonary hypertension, disruption of vascular integrity, and fibrosis [4].

Elimination attempts of vector-transmitted helminthic diseases have proven unsuccessful to date [5]. The challenges in eradicating *D. immitis* include controlling mosquito transmission, ensuring compliance with preventive measures among pet owners, and the emergence of resistance to macrocyclic lactones (ML). Currently, all Federal Drug Administration (FDA)-approved heartworm preventives for canines belong to the ML class of drugs, which includes ivermectin, selamectin, moxidectin, and milbemycin oxime [6]. Alternatively, an organic arsenical compound, melarsomine dihydrochloride, is the only available therapeutic approved by the FDA in dogs for the killing of adult heartworms [7]. Macrocyclic lactones are effective against L3 and L4 stages of *D. immitis* and dogs are susceptible to infection without regular administration of MLs [8]. There is a loss of efficacy concern with ML-resistant *D. immitis* cases increasing in the United States, and since most of the MLs require some aspect of host immunity to succeed at killing the L3 and L4, there is a need for a new class of drugs that is effective in killing *D. immitis* by either directly affecting the worm or enhancing the host immune response to the worm [6].

In addition to domestic dogs, *D. immitis* can establish in wild canids, cats, ferrets, and marine mammals, and can even infect humans [2]. Current in vivo *D. immitis* research relies on an established infection in a permissive host, and the best animal models for these studies are dogs and ferrets. Rodent animal models are preferred by researchers for in vivo work due to the nature of the research and economics; however, previous studies have attempted to establish infection in immunocompetent mice, rats, and jirds with variable success [7].

The Mongolian gerbil, *Meriones unguiculatus* (jird) serves as an immunocompetent rodent model for another filarial parasite, *Brugia malayi*, a causative agent of lymphatic filariasis [9]. Jirds develop a patent infection after subcutaneous (SC) and intraperitoneal (IP) inoculation of *B. malayi* L3 and developed adult worms are later found in the testes, heart, and lungs with SC infections and the peritoneal cavity in IP infections [9,10]. Additionally, microfilariae are found circulating in the peripheral blood of SC infections and peritoneal cavity of IP infections [9,10]. The IP-infected jird model is the ideal control for this study because of the ability to recover all developmental stages of the parasite from a single location [10].

**Figure 1:**
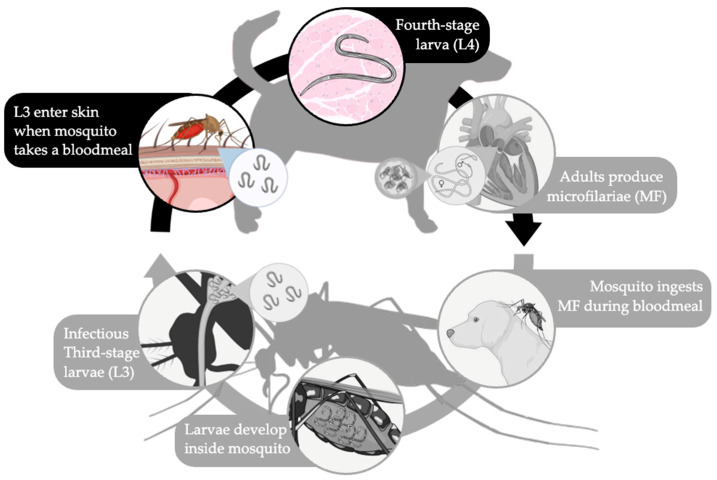
Hypothesis of when in the life cycle of *D. immitis* that host specificity occurs. We hypothesized that the establishment of *D. immitis* is determined by host immunity after the third larval molt (L3 to L4). Created with BioRender.com.

In this study, we observe the initial immune response of a nonpermissive host, the jird [11], to *D. immitis* IP infection. The objective of this study was to investigate the cellular immune response to infection with *B. malayi* and *D. immitis* larvae in the peritoneal cavity of the jird. We hypothesized that the establishment of *D. immitis* is determined by host immunity after the third larval molt (Figure 1). Identifying the *D. immitis* larval stage for which establishment is prevented by the jird nonpermissive host will provide more insight into host specificity of filarial infections.

## 2. Materials and Methods

### 2.1. Animals and Ethics Statement

All experiments with adult male jirds (*Meriones unguiculatus*) and purpose-bred cats and dogs were performed in accordance with the University of Georgia Institutional Animal Care and Use Committee guidelines and approved Animal Use Protocol A2022 04-009.

### 2.2. Parasites

*Brugia malayi* and *D. immitis* L3 were collected [12] from laboratory-raised *Aedes aegypti* black-eyed Liverpool mosquitoes [13] 15 days after feeding [14] on microfilaremic cat (Filariasis Research Reagent Resource Center, University of Georgia, Athens, GA, USA) and dog (University of Georgia, Athens, GA, USA) blood, respectively. Third-stage larvae were isolated into groups of 100 in Hanks’ balanced salt solution (HBSS; MP Biomedicals, Santa Ana, CA, USA) supplemented with 2% ciprofloxacin (Sigma-Aldrich, St. Louis, MO, USA).

### 2.3. Infection of Jirds

**Figure 2:**
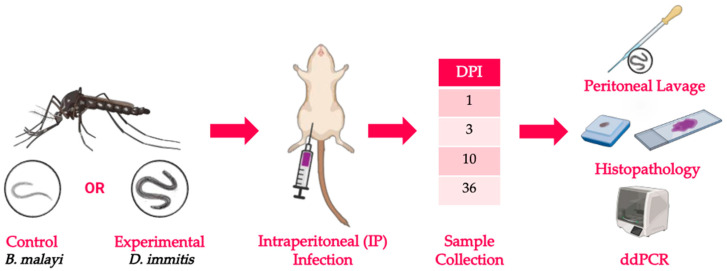
Experimental design. *Aedes aegypti* mosquitos were fed *B. malayi* and *D. immitis* microfilaremic blood, respectively. L3 were harvested from the mosquitos 15 days post blood feeding, and jirds were infected by IP injection with 100 *B. malayi* (*n* = 20) or *D. immitis* (*n* = 20) L3. Necropsies were performed at 1, 3, 10, and 36 days post infection (dpi) (*n* = 5/timepoint/group) according to the larval stage of the parasite. Larvae were recovered by peritoneal lavage at each timepoint, quantified, and stored in formalin for larval-stage morphologic identification. The peritoneal wall and diaphragm were recovered from each jird and stored in ethanol for absolute quantification of larvae with ddPCR. Histopathology was performed on the peritoneal wall and diaphragm of jirds infected by IP injection with either 500 *B. malayi* (*n* = 6) or *D. immitis* (*n* = 6) L3. H&E stains were used on the tissue slides for larval detection, and immunohistochemistry was also performed using anti-Iba1 antibody for detection of macrophages. Created with BioRender.com.

Adult male jirds were infected by intraperitoneal injection according to standard procedures [15] with either 100 *B. malayi* (*n* = 20) or *D. immitis* L3 (*n* = 20) for which the jird is permissive and nonpermissive to parasitic establishment, respectively. The jirds were separated into one of four timepoint groups: 1, 3, 10, and 36 dpi (Figure 2). 

### 2.4. Peritoneal Recovery of Larvae

Jirds were humanely euthanized by carbon dioxide asphyxiation followed by cervical dislocation at 1, 3, 10, or 36 dpi [10]. Larvae were collected by peritoneal lavage via a 5 mm abdominal incision with approximately 32 mL of 0.9% isotonic saline solution (Sigma-Aldrich, St. Louis, MO, USA) to maintain the shape and size of the peritoneal exudate cells (PECs) [10]. Recovered larvae were quantified and transferred from the cell suspension to 10% neutral-buffered formalin (Epredia, Kalamazoo, MI, USA) for identification of the larval stage. All remarkable observations were documented, including PEC attachment to larvae and degenerated larvae. The peritoneal wall (separated into the subject-oriented right and left side) and diaphragm of each jird were excised and stored in 70% ethanol (Sigma-Aldrich, St. Louis, MO, USA) for further analysis with ddPCR.

### 2.5. Histopathology

Adult male jirds were infected by IP injection according to standard procedures [15] with either 500 *B. malayi* (*n* = 6) or *D. immitis* (*n* = 6) L3. A naïve jird (*n* = 1) served as a sham control for infection and was inoculated with parasite-free HBSS (MP Biomedicals, Santa Ana, CA, USA) supplemented with 2% ciprofloxacin (Sigma-Aldrich, St. Louis, MO, USA) for which the same procedures were performed using mosquitoes that had been fed uninfected dog blood 15 days prior. All jirds were humanely euthanized by carbon dioxide asphyxiation followed by cervical dislocation at 1 dpi. Following euthanasia, a peritoneal lavage was performed as previously described; larvae were recovered, quantified, and the developmental stages of the larvae were identified. The peritoneal wall (separated into the subject-oriented right and left side) and diaphragm of each jird were excised and stored in 10% neutral-buffered formalin (Epredia, Kalamazoo, MI, USA) and submitted to the University of Georgia Histology Laboratory (Athens, GA, USA) for processing, step sectioning at 5 µm thickness, 10 µm apart, and staining with hematoxylin and eosin (H&E) stains for larval detection. Immunohistochemistry was also performed by the University of Georgia Histology Laboratory with 1:8000 dilution of anti-ionized calcium binding adaptor molecule rabbit (Iba1; FUJIFULM Wako Chemicals, Richmond, VA, USA) for the detection of macrophages [16]. Light microscopic evaluation (Olympus BX41; Olympus America Inc., Center Valley, PA, USA) was performed on the slides and images were captured using Olympus CellSens software, version 1.16 (Olympus America Inc., Center Valley, PA, USA).

### 2.6. DNA Extraction for Conventional PCR Validation of Primers

Genomic DNA was extracted from the diaphragms of naïve jirds (*n* = 3) combined with 1, 50, and 100 *D. immitis* L3, respectively, using the REDExtract-N-Amp™ Tissue PCR Kit (Sigma-Aldrich, St. Louis, MO, USA) as per the manufacturer’s instructions. DNA was stored at 4 °C until PCR was performed the following day.

### 2.7. Conventional PCR Validation of Primers

A conventional PCR assay, followed by gel electrophoresis, was performed to detect 1, 50, and 100 *D. immitis* L3 homogenized with naïve jird diaphragm, respectively. The mitochondrial cytochrome oxidase c subunit I (cox1) gene of *D. immitis* was amplified using forward (5′-CAT CCT GAG GTT TAT GTT ATT TT-3′) and reverse (5′-CWG TAT ACA TAT GAT GRC CYC A-3′) primers (Integrated Data Technologies, Coralville, IA, USA) [17,18,19]. Cycling conditions included an initial denaturation at 94 °C for 3 min, followed by 35 cycles for 1 min of denaturation at 94 °C and annealing at 45 °C, extension at 72 °C for 30 s, and a final extension at 72 °C for 1 min. The jird highly expressed reference gene, glyceraldehyde-3-phosphate dehydrogenase (GAPDH) (forward: 5′-CAT GGC CTT CCG AGT TCC T-3′; reverse: 5′-TTC TGC AGT CGG CAT GTC A-3′) (Integrated Data Technologies, Coralville, IA, USA), served as a positive control [20,21,22]. Cycling conditions included an initial denaturation at 94 °C for 3 min, followed by 35 cycles for 1 min of denaturation at 94 °C and annealing at 51 °C, extension at 72 °C for 30 s, and a final extension at 72 °C for 1 min. All reaction samples were performed in 50 µL volumes using 0.4 µM of each primer, 25 µL REDExtract-N-Amp™ PCR Reaction Mix (Sigma-Aldrich, St. Louis, MO, USA), and 200 pg/µL DNA template. Electrophoresis (Fisherbrand Mini-Horizontal Electrophoresis Systems, Fisher Scientific, Hampton, NH, USA) was performed in a 2% agarose 1x TAE gel stained with ethidium bromide (Sigma-Aldrich, St. Louis, MO, USA), and the PCR products were visualized under a UV light.

### 2.8. DNA Extraction for Droplet Digital PCR (ddPCR)

DNA was extracted from the peritoneal wall (separated into the subject-oriented right and left side) and diaphragm of each jird (*n* = 10), collected only at the 1 dpi timepoint. The tissue from each jird was removed from the 70% ethanol in which they were stored, followed by liquid nitrogen homogenization with mortar and pestle. All the peritoneal wall and diaphragm tissue collected from the jirds were weighed (a mean of 193 mg of diaphragm and 859 mg each peritoneal wall) and processed using the QIAGEN Blood & Cell Culture DNA Maxi Kit and QIAGEN Genomic-tips (QIAGEN Sciences Inc., Germantown, MD, USA) as per the manufacturer’s instructions for DNA extraction. Precipitation of DNA was performed to concentrate each sample as recommended by QIAGEN Sciences, Inc. A 1/10 volume of 3 M sodium acetate (pH 5.2) and 2 volumes of ice-cold 100% ethanol was added to the DNA sample. The sample was mixed and stored at −20 °C for 1 h to precipitate the DNA. The precipitated DNA was recovered by centrifugation at 21,000× *g* in a microcentrifuge for 20 min. The ethanol was discarded by rapid decantation and the pellet washed twice with room-temperature 70% ethanol. The DNA pellet was air-dried prior to resuspension in 100 µL distilled water. Quality control of the DNA samples was performed on a NanoDrop™ 2000 Spectrophotometer (Thermo Fisher Scientific, Waltham, MA, USA). DNA was stored at −80 °C until shipped to Emory Integrated Genomics Core for ddPCR.

### 2.9. Droplet Digital PCR (ddPCR)

The absolute quantification of *B. malayi* and *D. immitis* L3 DNA in the jird peritoneal wall and diaphragm was performed on a Bio-Rad QX200 w/ADG ddPCR System (Bio-Rad Laboratories, Hercules, CA, USA) by the Emory Integrated Genomics Core. This system of digital PCR uses water–oil emulsion droplet technology combined with fluidics [23]. A DNA sample, along with primers, probes, and reaction mix (2x ddPCR™ Supermix for Probes (no dUTP) (Bio-Rad Laboratories, Hercules, CA, USA) was divided into 20,000 nanoliter-sized droplets in which the DNA was randomly distributed, followed by PCR amplification of the DNA in each individual droplet [23].

The mitochondrial cytochrome oxidase c subunit I (cox1) gene of *D. immitis* was amplified using forward (5′-CAT CCT GAG GTT TAT GTT ATT TT-3′) and reverse (5′-CWG TAT ACA TAT GAT GRC CYC A-3′) primers with a FAM-fluorescent probe (5′-/56-FAM/CGG TGT TTG/ZEN/GGA TTG TTA GTG/3IABkFQ/-3′) (Integrated Data Technologies, Coralville, IA, USA) [17,18,19]. Cycling conditions included an initial denaturation at 94 °C for 3 min, followed by 35 cycles for 1 min of denaturation at 94 °C and annealing at 45 °C, extension at 72 °C for 30 s, and a final extension at 72 °C for 1 min. *Brugia malayi* HhaI, the most repeated gene in the genome, was amplified using forward (5′-GCA ATA TAC GAC CAG CAC-3′) and reverse (5′-ACA TTA GAC AAG GAA ATT GGT T-3′) primers with a FAM-fluorescent probe (5′-/56-FAM/TTA GTA GTT/ZEN/TTG GCA CTT/3IABkFQ/-3′) (Integrated Data Technologies, Coralville, IA, USA) [24,25]. Cycling conditions included an initial denaturation at 94 °C for 3 min, followed by 35 cycles for 1 min of denaturation at 94 °C and annealing at 45 °C, extension at 72 °C for 30 s, and a final extension at 72 °C for 1 min.

Completing the ddPCR process, each droplet was read by a droplet reader to determine the fraction of positive droplets in the total DNA sample, using Poisson statistical formulas to determine absolute quantification without the need for a standard curve [23].

### 2.10. Data Analysis

The sample size for this study was based on a study power of 0.8 and significance level of 0.05 with G*Power 3.1.9.7 software for Windows (Heinrich Heine Universität Düsseldorf, Düsseldorf, Germany). A contingency analysis was performed on recovered *D. immitis* and *B. malayi* larvae at each timepoint, respectively, from the total amount of L3 administered to the jirds. A Chi-square two-sided test followed the contingency analysis. Larval recovery analyses were performed with GraphPad Prism version 10 (GraphPad Software, San Diego, CA, USA). Copy numbers of *D. immitis* cox1 gene and *B. malayi* HhaI gene will be determined by ddPCR and analyzed with Poisson correction and equation on QuantaSoft™ Analysis Pro software version 1.4 (Bio-Rad Laboratories, Hercules, CA, USA). 

Descriptive observational analyses of the histopathological slides were performed by a boarded veterinary pathologist blinded to the samples’ infection status and parasite using a semi-quantitative scoring system to categorize the intensity of the cellular response. Low (+) intensity was defined as small, loose aggregates of inflammatory cells focused primarily around the parasite. Moderate (++) intensity was defined as larger, multifocal aggregates or mats of cells that expanded the perimysium or endomysium. This relative scale allowed for a consistent comparison of the inflammatory landscapes between the permissive control and nonpermissive experimental groups.

## 3. Results

### 3.1. Larval Recovery and Cell Attachment

**Figure 3:**
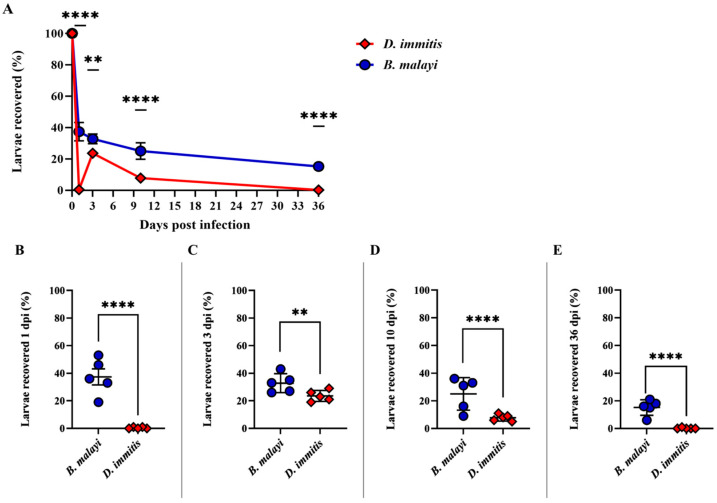
*D. immitis* and *B. malayi* larvae recovered from the peritoneal cavity of the Mongolian gerbil: (**A**) The percentage of larvae recovered from *B. malayi*-infected jirds (mean: 37.4%) after 1 day was significantly greater (*p* < 0.0001) than larvae recovered from *D. immitis*-infected jirds (0.4%). There was a reappearance of *D. immitis* larvae recovered at 3 dpi (23.6%; *p* < 0.001). (**B**–**E**) *D. immitis* larvae recovered from the peritoneal cavity on days 1, 3, 10, and 36 dpi were significantly fewer than *B. malayi* larvae (*p* < 0.001 3 dpi; *p* < 0.0001 1, 10, and 36 dpi). **: *p* < 0.001; ****: *p* < 0.0001.

A mean of 37.4% (SEM = 5.8%) *B. malayi* L3 and 0.4% (SEM = 0.2%) *D. immitis* L3 were recovered at 1 dpi (*p* < 0.0001). *Brugia malayi* L3 recovery remained the same (mean = 32.8%; SEM = 3.1%) and a mix of *D. immitis* L3 and L4 were recovered (23.6%; SEM = 1.8%) at 3 dpi, a significant increase from 1 dpi for *D. immitis* (*p* < 0.001). Both *B. malayi* (mean = 25%; SEM = 5.3%) and *D. immitis* L4 recovery (mean = 7.8%; SEM = 1.1%) steadily decreased at 10 dpi (*p* < 0.0001). By 36 dpi, a mean of 15.2% (SEM = 2.5%) *B. malayi* immature adults (IA) and no viable *D. immitis* larvae were recovered (*p* < 0.0001) (Figure 3). In summary, *D. immitis* larvae are not recovered by peritoneal lavage at 1 dpi infection, reappear in the peritoneal cavity at 3 dpi, all viable larvae are L4 by 10 dpi, and larvae are no longer recovered in the peritoneal cavity by 36 dpi.

**Figure 4:**
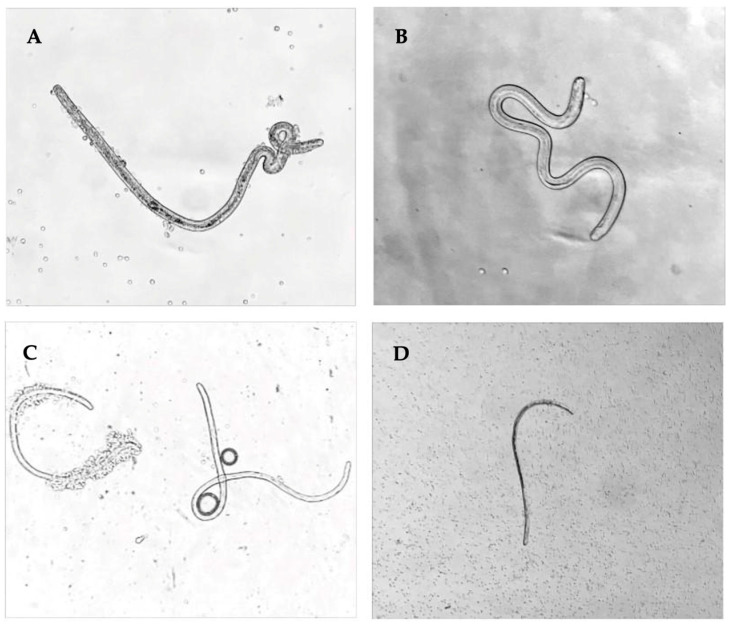
Peritoneal exudate cells attach to *D. immitis* L3. *D. immitis* larvae were recovered from the peritoneal cavity at 1, 3, 10, and 36 dpi. PECs were attached to *D. immitis* L3 at 3 and 10 dpi: (**A**) some *D. immitis* L3 had PEC attachment at 3 dpi; (**B**) a shed cuticle from a *D. immitis* L3 was observed 3 dpi after its third molt; (**C**) at 10 dpi, a *D. immitis* L3 with PEC attachment and an L4 with no cell attachment was observed; and (**D**) the only *D. immitis* larvae recovered at 36 dpi was degenerated.

The authors observed jird peritoneal exudate cell (PEC) attachment to *D. immitis* larvae recovered in peritoneal lavage fluid at 3 and 10 dpi and observed only one degenerated larva at 36 dpi (Figure 4). The observed cell attachment to *D. immitis* larvae in vivo complements the results previously seen in vitro [16].

### 3.2. Histopathology of the Peritoneal Wall and Diaphragm

The peritoneal wall and the diaphragm of a naïve jird, Jird A, served as a negative control to establish a baseline cellular response. Jird A’s right and left peritoneal wall contained focal, mild satellite cell proliferation, suggesting muscle regeneration. The left peritoneal wall contained locally extensive infiltration of the perimysium, which surrounds bundles of muscle fibers, by moderate numbers of neutrophils and macrophages (Table 1), extending into the muscle, effacing a few myofibers, and dissecting between others. A thin strip of dense, diaphragm connective tissue was multifocally infiltrated by moderate numbers of neutrophils, lymphocytes, and macrophages, particularly surrounding vessels, with another focal aggregate of inflammatory cells adhered to the skeletal muscle surface.

**Figure 5:**
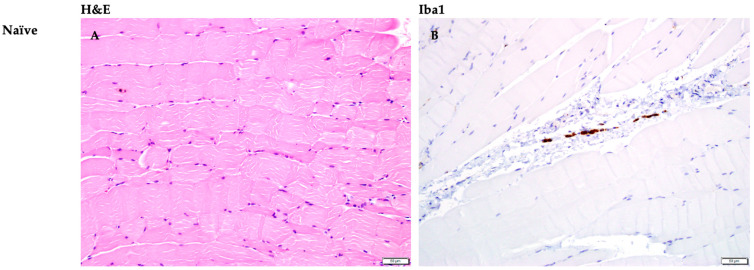

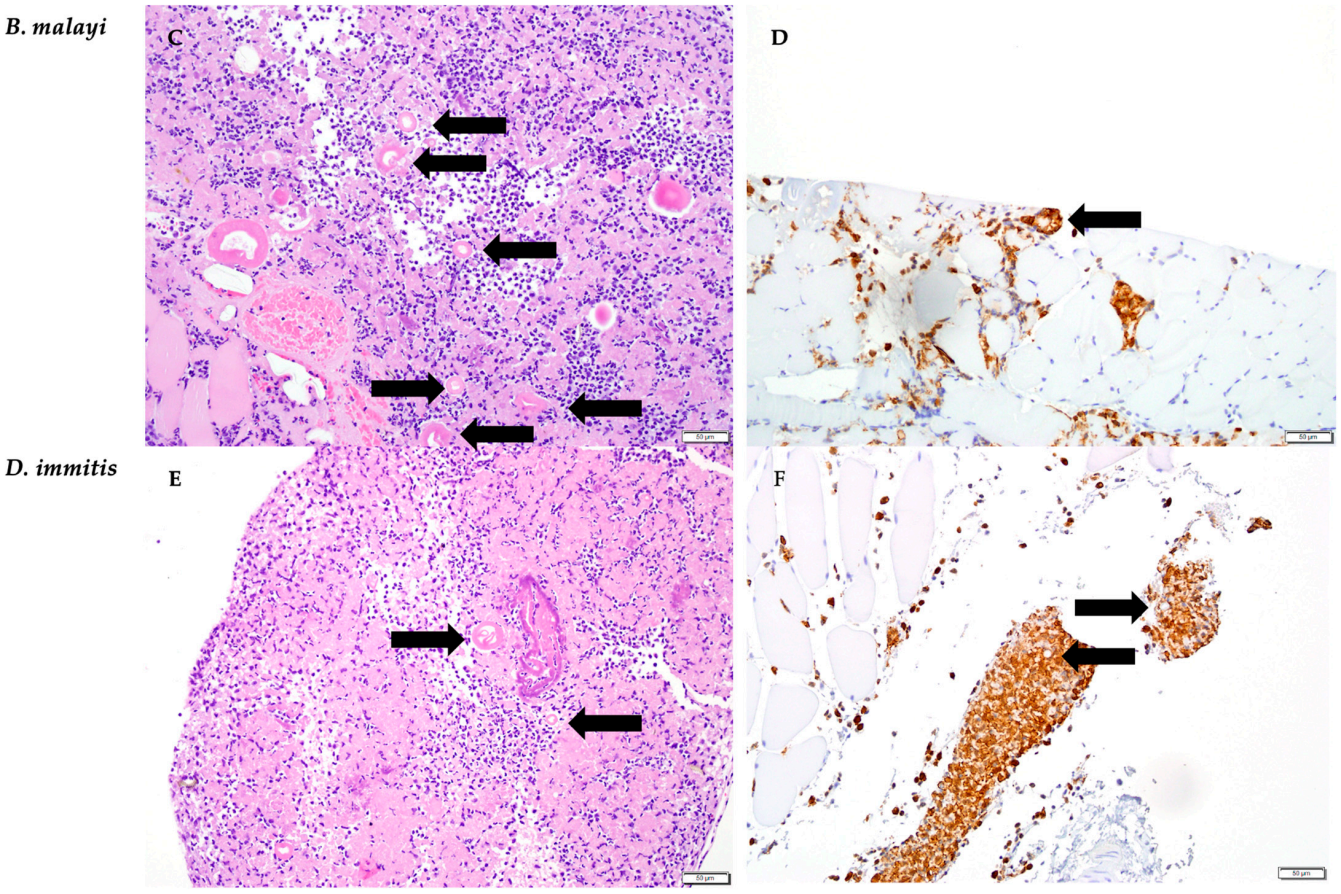
Larvae sequestered in the peritoneal wall and diaphragm of the jird. All images are of jird peritoneal wall. Panels (**A**,**C**,**E**) tissues were stained with H&E, staining the leukocytes dark purple. Panels (**B**,**D**,**F**) tissues were stained for Iba1 using immunohistochemistry, showing macrophages as brown (immunopositive). Arrows direct attention to larvae embedded in tissue.

Although six animals per group were infected with 500 larvae, histopathology focus was placed on those where larvae were successfully located in tissue sections: Jirds B, C, and D where the larvae were definitively found. The most notable results in *B. malayi*-infected jirds came from two jirds, Jird B and Jird C (Table 1). The right and left peritoneal wall and diaphragm of Jird B contained locally extensive aggregates of neutrophils, macrophages, and lymphocytes with fibrin, necrotic debris, and entrapped, variably sized, degenerated nematodes. Degenerated nematodes (Figure 5) were also found lining the surface of the diaphragm and along a strip of associated connective tissue. Both the right and left peritoneal wall had perimysium and interstitium that were multifocally expanded by edema and small numbers of neutrophils, lymphocytes, and macrophages. Similarly, the right peritoneal wall of Jird C contained multifocal mats of fibrin, mixed with neutrophils, lymphocytes, and macrophages, along the surface of the epimysium, a connective tissue sheath surrounding muscle. The perimysium and epimysium were multifocally expanded by edema and low (right peritoneal wall) to moderate (left peritoneal wall and diaphragm) numbers of neutrophils, macrophages, and lymphocytes, with scattered degenerated and regenerative myofibers. The diaphragm of Jird C had multifocal mats of neutrophils, macrophages, and lymphocytes, with fibrin and necrotic debris, lining the surface of the muscle and the connective tissue layer with a viable larva entrapped in exudate.

Notably, the right and left peritoneal wall and diaphragm of Jird D (Table 1), infected with *D. immitis*, were multifocally lined by of neutrophils, macrophages, and lymphocytes, with fibrin and necrotic debris, on the muscle surface. The perimysium and endomysium were multifocally expanded by edema and low to moderate numbers of neutrophils, lymphocytes, and macrophages with scattered degenerated and regenerative myofibers. A few degenerate and viable larvae were observed in the left peritoneal wall, and a single, entrapped, larval nematode lining the muscle surface and connective tissue layer was observed in the diaphragm (Figure 5).

To further characterize the cellular response, immunohistochemistry was performed for Iba1 (Figure 5). In both *B. malayi* and *D. immitis* infected tissues, Iba1-positive macrophages were identified as the predominant cell type within the inflammatory mats and specifically surrounding sequestered larvae. This was characterized by intense brown (immunopositive) staining of macrophages localized around both viable and degenerated nematodes.

## 4. Discussion

There is a lack of knowledge regarding the host–parasite interaction during *D. immitis* infection, specifically the mechanisms of the canine host that determine the ultimate establishment of the parasite. A comprehensive understanding requires a thorough investigation into each life stage to understand not only the mechanisms of worm establishment within the host, but also their interaction with the host during establishment. Due to the limited availability of *D. immitis* in research settings, most in vitro studies focus on the MF and L3/L4 life stages of the parasite, which often leads to a knowledge gap regarding the mechanisms of the host that determine parasite establishment in vivo. As the threat of ML-resistant *D. immitis* rises in the United States, there is a need for either a new class of drugs that is effective in killing *D. immitis*, or another target of host immunity to explore to work in tandem with MLs [6].

In this study, the authors explored the determinants of *D. immitis* host specificity using a nonpermissive rodent model, the Mongolian jird (*Meriones unguiculates*). We hypothesized that host immunity determines the establishment of *D. immitis* after the third larval molt. The data presented here partially support this hypothesis, specifically indicating that establishment occurs following the third larval molt. While the absence and reappearance of larvae at 1 and 3 dpi, respectively, suggest a period of transient tissue localization, we acknowledge that technical recovery losses or early larval mortality remain alternative possibilities. However, the recovery of viable L4 at 3 dpi suggests sequestration is the most probable scenario.

Only one publication documents the development of intraperitoneal infection of *D. immitis* in the jird [11]. Wong and Lim inoculated jirds with large numbers of L3 IP (400–600), and worms were recovered between 16 and 132 dpi, the earliest timepoints yielding minimal recovery: 0.5% L4 at 16 dpi, 3.5% L4 at 23 dpi, and 0.75% L4 at 44 dpi. This current study infected jirds with fewer (*n* = 100) L3 than in that study, but the results were similar: *D. immitis* larvae did not develop past the L4 stage except for one mating pair of adult worms that was recovered (*n* = 1 of 5 jirds) alive from the pulmonary artery and right heart [11]. We did not maintain our gerbils for a sufficient amount of time required to determine whether there was development into adults.

*Dirofilaria immitis* L3 were found in the peritoneal wall and diaphragm of jirds, which were examined for their cellular response to infection with *B. malayi* and *D. immitis* L3. A naïve jird served as a negative control for the cellular response; the peritoneal wall had focal, mild satellite cell proliferation, suggesting muscle regeneration, and only the left peritoneal wall had extensive infiltration of perimysium by a moderate number of neutrophils and macrophages. This was a deliberate sham procedure designed to evaluate the baseline inflammatory response elicited by the injection of the media from the specific L3 harvest process. Neutrophils were observed as early responders in both *B. malayi* and *D. immitis* infections. Their presence likely represents an innate response to the “sequestration”, or the initial mechanical presence of the L3, potentially serving as the first line of defense that facilitates the recruitment of more specialized cells [7,16]. Although the naïve jird was not infected with larvae, the peritoneal cavity was inoculated with nonsterile HBSS that was subjected to the same collection procedures as the experimental groups. Because this solution likely contained microscopic mosquito debris, it served as an intended sham to distinguish between inflammation caused by procedural artifacts and the specific response triggered by the parasites.

The jirds for histopathology received a higher inoculum (500 L3) to increase the probability of locating larvae within tissue sections (essentially searching for a “needle in a haystack”); we suspect this larger volume of foreign material may have increased the speed and intensity of the immune response compared to the 100 L3 dose used in the recovery studies. Nevertheless, jirds infected with *D. immitis* displayed multifocal mats of neutrophils, macrophages, and lymphocytes with fibrin and necrotic debris, in contrast with the loose aggregates observed in *B. malayi* tissues. We hypothesize that these macrophage-rich infiltrates, confirmed via Iba1 IHC, play a central role in preventing parasite establishment. The presence of Iba1-positive macrophages forming these mats suggests they are the primary effector cells responsible for parasite arrest in the nonpermissive host. These macrophages likely participate in adhering to the larval cuticle and releasing lysosomal enzymes to clear the parasite [4,7]. Our previous observations of jird PEC attachment in vitro [16] support this role, suggesting an aggressive and organized attack specifically against *D. immitis* compared to the permissive development seen in the dog [3,26] or the *B. malayi* jird model [9,10].

Furthermore, the presence of lymphocytes within these inflammatory mats indicates the activation of the early adaptive immune response, likely orchestrating the inflammatory landscape by secreting cytokines that polarize the macrophage response toward clearance [27]. While lymphocytes were identified morphologically, it was not possible to definitively subtype these cells (e.g., T-cell or B-cell subpopulations) in this study. This is a recognized limitation of the Mongolian jird model, as there is currently a significant lack of validated, commercially available, jird-specific cluster of differentiation (CD) markers for immunohistochemistry [22].

The most notably affected *B. malayi* jirds contained extensive aggregates of neutrophils, macrophages, and lymphocytes with fibrin, necrotic debris, and degenerated nematodes. Compared to the naïve jird, there was an increase in the number of areas with immune cells, and these areas were focused around the degenerated larvae. The cellular immune response observed in the jird to *B. malayi* aligned with the cell types observed in previous studies with subcutaneously infected jirds. These studies observed a moderate number of macrophages, a few lymphocytes and neutrophils, and a few eosinophils as early as 1 and 7 dpi, respectively [27,28].

Observing a cellular immune response in the naïve jird negative control and *B. malayi* positive control serves as a baseline for evaluation of the jird cellular response to *D. immitis* L3. Jirds infected with *D. immitis* had multifocal mats of neutrophils, macrophages, and lymphocytes, with fibrin and necrotic debris on the muscle surface in contrast with the loose aggregates of cells observed in *B. malayi* tissues. The perimysium and endomysium were expanded by edema, with low to moderate numbers of similar inflammatory cells. Although the negative control displayed perimysial infiltration by macrophages and neutrophils, likely due to the sham HBSS at time of inoculation, the *D. immitis* tissue additionally contained lymphocytes and edema of the perimysium. Overall, there was an increase in inflammation localized around *D. immitis* L3 compared to *B. malayi*-infected peritoneal wall and naïve tissue.

Peritoneal recovery of *D. immitis* larvae in the jird was as expected, with a gradual decline, but the absence and reappearance of *D. immitis* larvae at 1 and 3 dpi, respectively, was of the utmost interest to the authors. Although the recovery of *B. malayi* larvae was slightly lower at 10% than the expected 23%, this could be due to the overall variability of jird infectivity [29]. Previous studies demonstrated that subcutaneously inoculated *D. immitis* L3 and L4 are found in the skin and muscle of dogs and ferrets up to 12 and 58 dpi, respectively [26,30], so the peritoneal wall and diaphragm were excised and submitted for histopathology to potentially locate L3 in the tissues at 1 dpi. Although *D. immitis* L3, along with a few *B. malayi* L3, were observed in the step sections of these tissues, it was highly likely that larvae were overlooked through the process. Notably, there was a clear increase in the presence of inflammatory cells and macrophages that appeared in the tissues with *D. immitis* L3 as compared to the tissues with *B. malayi* L3. These results demonstrate that there is a cellular immune response to *D. immitis* L3 and *B. malayi* L3.

In 2021, Evans et al. reported jird peritoneal exudate cell attachment in vitro to *D. immitis* L3 after 20 h of culture [16]. The cells attaching to *D. immitis* L3 were identified primarily as macrophages. Similarly, this study observed cell attachment to viable *D. immitis* L3 at 3 dpi (Figure 5). It is likely that the PECs attached to L3 in the peritoneal cavity are primarily macrophages, considering that the primary cell type surrounding larvae in the tissues is the macrophage as seen in the Iba1 IHC slides (Figure 5).

A major limitation of this research is the jird model. There are almost no immunochemistry markers known to work with gerbils, and characterization of jird peritoneal cells has yet to be published. There are more unknowns than knowns regarding gerbil immunity, requiring researchers to gain a more basic understanding of jirds before dedicating their efforts to using them as a model. However, the jird was an ideal model for this study because it is permissive to the establishment of a *B. malayi* intraperitoneal infection, allowing for an easier method of worm recovery than a subcutaneous inoculation method.

The histopathology results were useful in describing the cellular immune response in the peritoneal wall and diaphragm of the parasites. However, through the process of sectioning samples, the probability of overlooking larvae is high. Droplet digital PCR is a more sensitive test for the absolute quantification of DNA than other PCR methods. By quantifying the copy numbers of parasite DNA within each tissue sample, the issue of potentially missing L3 with light microscopy should be resolved.

However, the results of the ddPCR performed in this study were inconclusive. The purpose of performing the ddPCR was to use a more sensitive method of detecting L3 in the tissues instead of histopathology since the latter method was similar to trying to find a “needle in a haystack”. The mass of the tissue sections (mean = 859 mg for the wall and 193 mg for the diaphragm) was quite large compared to a single L3, and it is likely that host DNA overpowered the parasite DNA during extraction. Technical analysis revealed that concentration values for infected samples (0.26 to 14.74 copies/µL) were often indistinguishable from the background noise observed in jird-only controls (0.53 copies/µL). While spiking experiments successfully validated our cox1 primers for conventional PCR, the low copy numbers in L3-only positive controls (0.39–0.84 copies/µL) indicate that host tissue interference significantly hindered the sensitivity required for definitive quantification. Additionally, several samples failed to produce droplets for the *D. immitis* cox1 and *B. malayi* HhaI assays. Despite these technical hurdles, this study demonstrates that the nonpermissive host has an increased cellular immune response to *D. immitis* compared to *B. malayi*.

It is well known that macrophages target extracellular parasites by alternative activation, so investigation into the specific role macrophages play in preventing the establishment of *D. immitis* in the jird is necessary to better understand host specificity from the perspective of host–parasite interactions. This is the first study to document the initial immune response of the jird to intraperitoneal inoculation with *D. immitis* L3. The majority of *D. immitis* research is performed in vitro due to the limited availability of resources and culture surrounding animal research [31,32,33,34]. It is essential to shift perspectives and explore new avenues to understand the host–parasite interaction in the initial stages of infection. This study demonstrates that the nonpermissive host has an increased cellular immune response to *D. immitis* compared to *B. malayi*, for which the jird is susceptible to infection. Since MLs work with the host immune system to prevent establishment and eliminate *D. immitis*, the jird cellular immune response to *D. immitis* should be further explored.

## 5. Conclusions

Current *D. immitis* research primarily relies on established infections in permissive hosts, such as dogs and ferrets. This study, however, is the first to investigate the initial immune response of a nonpermissive host, the jird, to *D. immitis* intraperitoneal infection, with results supporting the hypothesis that host immunity determines the establishment of *D. immitis* after the third larval molt. The most notable finding was the temporary absence and reappearance of *D. immitis* larvae in the peritoneal cavity between 1 and 3 days post infection (dpi), as larvae were found sequestered in the peritoneal wall and diaphragm at 1 dpi, surrounded by an increased cellular immune response comprised primarily of macrophages, followed by neutrophils and lymphocytes. Identifying the larval stage at which *D. immitis* establishment is prevented by the nonpermissive jird host is crucial, as this insight into host specificity and the early host–parasite interaction can inform the development of new drugs or host immunity targets to combat the increasing threat of ML-resistant *D. immitis* in the United States.

## Figures and Tables

**Table 1:** Cells observed surrounding larvae in the peritoneal wall and diaphragm of the jird. A naïve jird served as a control for the cells seen in infected tissues. *B. malayi* L3 was observed in only two of the five infected jirds. *D. immitis* L3 was observed in only one of the five infected jirds.

Jird	Parasite	L3 Observed	Predominant Cell Types	Location of Response
A	Naïve	None	Neutrophils, Macrophages	Perimysium (Left Wall/Diaphragm)
B	*B. malayi*	Degenerated (++)	Neutrophils, Macrophages, Lymphocytes	Right/Left Wall, Diaphragm
C	*B. malayi*	Viable (+)	Neutrophils, Macrophages, Lymphocytes	Diaphragm
D	*D. immitis*	Viable (++) Degenerated (++)	Neutrophils, Macrophages, Lymphocytes	Right/Left Wall, Diaphragm

+ Denotes a single nematode; ++ Denotes < 10 nematodes. Fibrin and necrotic debris were present in all infected jird tissues.

## Data Availability

The data presented in this study are available on request from the corresponding author.

## Abbreviations

The following abbreviations are used in this manuscript:

CD	Cluster of differentiation
cox1	Cytochrome oxidase c subunit I
ddPCR	Droplet digital PCR
DNA	Deoxyribonucleic acid
dpi	Days post infection
FDA	Federal Drug Administration
FR3	Filariasis Research Reagent Resource Center
GAPDH	Glyceraldehyde-3-phosphate dehydrogenase
H&E	Hematoxylin and eosin
HBSS	Hank’s balanced salt solution
IA	Immature adults
Iba1	Ionized calcium binding adaptor molecule 1
IHC	Immunohistochemistry
IP	Intraperitoneal
L3	Third-stage larvae
L4	Fourth-stage larvae
MF	Microfilariae
ML	Macrocyclic lactone
n	Sample size
PCR	Polymerase chain reaction
PEC	Peritoneal exudate cell
SC	Subcutaneous
SEM	Standard error of the mean
TAE	Tris-acetate-EDTA
UV	Ultraviolet
